# Metavirome Insights into the Diversity and Potential Pathogenic Infection of *Chlamys farreri* in the Coastal Seas of the Republic of Korea

**DOI:** 10.3390/pathogens13110935

**Published:** 2024-10-27

**Authors:** Ji Woo Shin, Kang Eun Kim, Joon Sang Park, Min-Jeong Kim, Taek-Kyun Lee, Yu Jin Kim, Hyun-Jung Kim, Seon Min Kim, Seung Won Jung

**Affiliations:** 1 Library of Marine Samples, Korea Institute of Ocean Science & Technology, Geoje 53201, Republic of Korea; sjw3003@kiost.ac.kr (J.W.S.); rkddmssl@kiost.ac.kr (K.E.K.); jspark1101@kiost.ac.kr (J.S.P.); min-jeong214@kiost.ac.kr (M.-J.K.); rladbwls06069@kiost.ac.kr (Y.J.K.); hjkim8845@kiost.ac.kr (H.-J.K.); seonmin@kiost.ac.kr (S.M.K.); 2 Department of Ocean Science, University of Science & Technology, Daejeon 34113, Republic of Korea; 3 Risk Assessment Research Center, Korea Institute of Ocean Science & Technology, Geoje 53201, Republic of Korea; tklee@kiost.ac.kr; 4 Department of Oceanography and Marine Research Institute, Pusan National University, Busan 46241, Republic of Korea

**Keywords:** aquaculture, *Chlamys farreri*, DNA virome, herpesvirus, *Ostreid herpesvirus 1*, viral diversity, seawater

## Abstract

*Chlamys farreri* is primarily cultivated in Japan, China, and South Korea. Although mass mortality of scallops has been occurring recently, likely caused by high temperatures or infectious diseases, the underlying cause remains unclear. Little is known regarding the viral diseases affecting them. Therefore, we explored DNA virus diversity in the mid-gut gland of *C. farreri* and compared it with that of seawater. *C. farreri* was cultivated at depths below 5 m from the sea surface in the coastal waters of South Korea and sampled from May to August 2018. Different DNA viral communities were observed in both *C. farreri* and seawater. In *C. farreri*, prevalent groups included *Mimiviridae* (7%), *Poxviridae* (6%), and *Phycodnaviridae* (5%). Conversely, the dominant groups in seawater were *Autographiviridae* (20%), *Kyanoviridae* (12%), and *Zobellviridae* (10%). We identified *C. farreri*-specific viral communities and potentially infectious viruses, such as *Ostreid herpesvirus 1* and Abalone herpesvirus Victoria/AUS/2009. Furthermore, *C. farreri* acts as a reservoir for various viruses, which impact microbial community dynamics and disease transmission in marine ecosystems. Understanding these viral communities is crucial to protecting and restoring coastal ecosystems by highlighting their role in the transmission of potential avian- and bivalve-specific viruses.

## 1. Introduction

Bivalves, especially clams, mussels, and oysters, are crucial in global aquatic food production [1], and 2.1 million tonnes (USD 5.3 million) of scallops were cultured through marine aquaculture in 2016 [2]. However, changes in the environment and mass farming lead to mass mortality in coastal aquaculture, and the emergence of pathogens through anthropic activities is one of the primary causes of mortality. For example, mass mortality of scallops began in China in the 1980s and continued for several years in the 1990s [3,4]. The bivalve shellfish farming industry has experienced enormous economic damage owing to various diseases. For example, infection with Herpes-like viruses has been reported in multiple marine bivalve molluscs worldwide [5,6,7,8,9], and mass mortality has recently increased in the Republic of Korea and China [10,11].

*Chlamys farreri* [12] is one of the largest cultured bivalves in north Asian coastal areas such as China, Japan, and Korea [13,14,15]. Various factors, such as viral infection, climate change, and physiological stress caused by mass farming, increase the mortality rate of *C. farreri* [16]. The mortality of shellfish is caused by various pathogens [17,18,19,20]. For example, Acute Viral Necrosis Virus, Scallop Picorna-like virus, Gill necrosis virus, Hemocyte infection virus, Oyster velar virus disease, and Haliotid herpesvirus-1 are known to infect bivalve organisms [21,22,23]. Herpesviruses are ubiquitously distributed worldwide and can infect marine bivalve molluscs. These viruses have adapted their infection mechanisms to a single host after a long period of co-evolution [24]; however, the host specificity of these viruses is unknown [25,26]. *Ostreid herpesvirus 1* (OsHV-1) belongs to the *Herpesviridae* family and can kill various bivalve molluscs such as oysters (*Crassostrea gigas*), scallops (*Chlamys farreri*), and clams (*Scapharca brokenonii*) [22,27,28,29]. Thus, these herpesviruses can cause mass scallop mortality because of viral bioaccumulation in the digestive glands through filter feeding [30]. However, research on how viruses in seawater differ based on their concentration in the digestive tract of filter-feeding bivalve molluscs is limited. The epidemiology of infectious viruses in bivalves was studied by (1) studying the viruses in ambient seawater to determine whether they are transmitted from scallop to other scallops and (2) studying changes in infectious viruses in the scallop. Therefore, the aim of this study was to elucidate the epidemiological importance of DNA viruses in the gastrointestinal microbiome of *C. farreri* by investigating the monthly changes in DNA viruses in scallops and the differences in the diversity of ambient seawater viruses through metagenomics.

## 2. Materials and Methods

### 2.1. Study Design and Sample Collection

**Figure 1:**
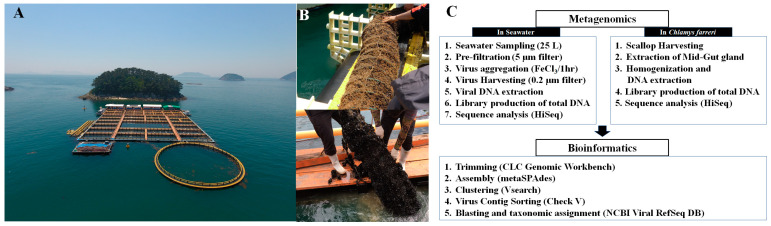
Photos of the field cultivation site of *Chlamys farreri* at the Tongyeong Megacosm Test Station at the Korea Institute of Ocean Science and Technology (**A**) and sampling photos of *C. farreri* acclimating to the lantern net in May (top photo) and August (down photo) (**B**). Flow chart illustrating the metavirome analysis process of seawater and *C. farreri* from sample collection to bioinformatics analysis (**C**).

In situ experiments were performed at the Tongyeong Megacosm test station (34°46′11.0″ N 128°22′40.6″ E) in the Southern Sea of South Korea (Figure 1). This *C. farreri* aquaculture area has clean water and a muddy and sandy bottom layer with a water depth of approximately 30 m. *C. farreri* were acclimatised in lantern nets at a depth of 5 m from the sea surface. Each net contained 60 individuals. The juveniles had an initial mean shell height of 3.7 ± 0.56 cm at the start of the experiment (January 2018). Culturing was performed without collection from February to April after initiating *C. farreri* farming in January, considering growth. Eight nets were installed each month (May to December 2018) to minimise the stress caused by air exposure when collecting *C. farreri*. After deployment, *C. farreri* were sampled monthly. The scallops were placed in plastic bags after the attached organisms and particles in the shell surface, such as mud, attached seaweeds, and other organisms, were removed. *C. farreri* were then stored in a portable freezer at −20 °C and transported to the laboratory. In the laboratory, the surface of the shell was washed with sterilised seawater, and the height and width of the shell were measured using vernier calliper (Table S1). The mid-intestinal glands (MGs), the digestive organs of *C. farreri*, from 15 individuals were pulverised using a homogeniser after freezing with liquid nitrogen to determine viral communities in the MGs of live *C. farreri*. In total, 30 individuals were obtained for triplicate experiments. The samples collected from the MGs were stored at −20 °C for no longer than two days. Ambient seawater samples at a depth of 5 m under the surface obtained from the cultivation sampling site were collected using a 10 L Niskin-type water sampler (General Oceanics, Miami, FL, USA) from May to December 2018. To analyse the DNA viral community, 25 L of seawater was collected for each experiment, and the experiments were performed in triplicate. The seawater was placed in sterile polypropylene bottles and stored in a 4 °C ice cooler until transported to the laboratory (Travel time: approximately 1 h). MGs were collected, and genomic DNA (gDNA) in the MG samples was extracted within two days. The EXO2 Multiparameter Sonde (YSI, Yellow Springs, OH, USA) measured water temperature and salinity at the sampling site. DNA viruses were harvested by collecting 25 L of seawater using FeCl_3_-based flocculation, filtration, and resuspension methods. To aggregate DNA viruses with Fe^3+^ ions, the mixture was maintained in a reservoir (cylindrical, made of black-coloured acrylic material) at 20 °C for 1 h. The DNA viruses aggregated with Fe^3+^ ions were collected onto a 0.2 μm polycarbonate membrane (Ca No. 111106; 47 mm; Whatman, Buckinghamshire, UK) for gDNA extraction from the DNA virus population (Figure 1C) [31,32]. The membranes were stored at 4 °C, and gDNA in membrane samples were extracted within two days.

### 2.2. Metavirome Analyses of Chlamys farreri and Seawater

The DNA metavirome analysis in seawater and scallops was performed by following our previous results [32]. The total viral gDNA from *C. farreri* and seawater was extracted using the Viral Gene-spin Viral DNA/RNA Extraction Kit (iNtRON Biotechnology, Seoul, Republic of Korea). Metagenomic libraries were constructed using the NEBNext Ultra II DNA Library Prep Kit (Illumina, San Diego, CA, USA) and sequenced using the Illumina HiSeq 2500 platform. The raw sequence data were trimmed to remove inappropriate sequences using CLC Genomics Workbench v. 20.0.4 (Qiagen, Hilden, Germany). Reads were assembled using metaSPAdes v. 3.13.0 [33], and viral contigs of >1000 bp were extracted from the total contigs using Check V v. 1.0.1 [34]. These viral contig sequences with >95% nucleotide identity were selected using VSEARCH [35]. Read mapping was performed with BBMap v. 38.51 using a 95% minimum alignment identity [36]. The quality-checked viral contigs were subjected to a virus taxonomy analysis using Basic Local Alignment Search Tool (BLASTn) analysis with the Microbial Genomic Module in the CLC Genomics Workbench with the Viral RefSeq database (Release 221) of the National Center for Biotechnology Information.

### 2.3. Statistical Analysis

The results are presented as the mean of replicate samples. Hierarchical cluster analysis and non-metric multi-dimensional scaling (NMDS) using Bray–Curtis dissimilarity were performed using PRIMER 6 (v 6.1.13) to elucidate the difference in the DNA viral community between *C. farreri* and seawater. A Venn diagram was created using the ggplot2 function [37] in R Studio to analyse the differences in DNA virus composition between seawater and scallops as well as those between different months of sample collection. A heatmap of the relative abundance of common DNA viruses (mean value: >0.1%) between *C. farreri* and seawater was created to visualise the differences using ggplot2. The Shannon diversity index was analysed using the vegan package (v 2.6-6.1) in R Studio. A Wilcoxon test was performed to examine the differences among groups of herpesviruses and *Ostreid herpesvirus 1*.

## 3. Results and Discussion

**Figure 2:**
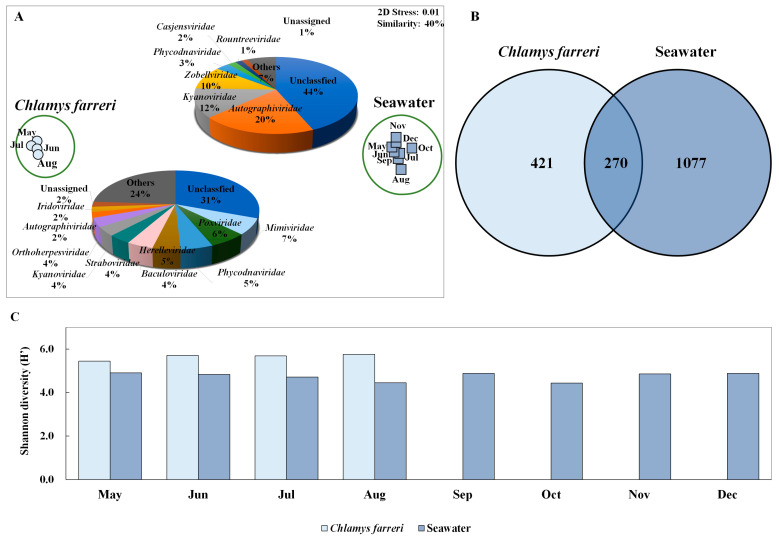
Non-metric multi-dimensional scale (NMDS) plot based on the results of Bray–Curtis dissimilarity analysis showing the diversity of the marine dsDNA virus community in *Chlamys farreri* and seawater (**A**). Pie charts depicting the high-ranking taxonomic distribution at the family level for the viral community. (**B**) Venn diagram showing the shared and unique total scallop and marine viral operational taxonomic units (vOTUs) (**C**). Shannon diversity index illustrating changes in dsDNA virus community in *C. farreri* and seawater.

Changes in the viral community were investigated through intestinal microbiome analysis along with the growth of *C. farreri* from May to December 2018, but all scallops died in September; therefore, the experiment was conducted until August (Figure 1B). However, the viral community in seawater was observed continuously until December. Taxonomic analysis of contigs in the genome fragments confirmed their identity as DNA viruses. In total, 1263 and 9525 DNA virus contigs were detected in *C. farreri* and seawater, respectively, through metavirome analysis (Table S2). The metagenomic analyses were based on triplicate experiments, but some samples were excluded during the study because they failed the quality check. NMDS analysis was used to classify the samples at a similarity of 40% between DNA viral communities of *C. farreri* and of seawater (Figure 2A). In seawater, the most highly occurring classified viral communities (at the family level) were *Autographiviridae* (mean: 20%), *Kyanoviridae* (12%), and *Zobellviridae* (10%). In comparison, viruses in *C. farreri* were primarily *Mimiviridae* (7%), *Poxviridae* (6%), and *Phycodnaviridae* (5%). These results indicate a remarkable difference in the viral community between seawater and *C. farreri*. In the Venn diagram, 270 viral operational taxonomic units (vOTUs) commonly appeared in seawater and *C. farreri*, 1077 vOTUs only appeared in seawater, and 421 vOTUs only appeared in *C. farreri* (Figure 2B). In addition, the diversity index of the viral community in scallops was 5.60, which was higher than that in seawater (4.66) (Figure 2C). The high viral diversity observed in scallops aligns with previous findings [22,38,39]. As filter feeders, scallops consume various eukaryotic planktons and microorganisms found in seawater, resulting in a heightened detection of virus types compared to that in seawater. Destoumieux-Garzón [40] reported that bivalves concentrate microorganisms from the surrounding seawater. The microbial community within bivalves is highly distinct from the microbiome found in the surrounding seawater.

**Figure 3:**
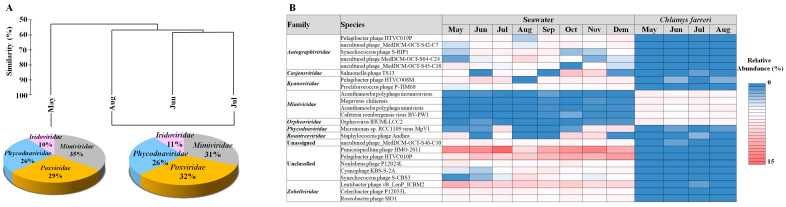
Hierarchical agglomerative clustering performed based on the group abundance of the *Chlamys farreri* (**A**). Pie charts indicate the high-ranking taxonomic distribution for the viral community. (**B**) Heatmap displaying the relative abundance of dominant taxa occurring at 1% or greater in the virus community.

In *C. farreri*, the viral community was divided into two groups of “in May” and “between June and August”. The major families in the two groups of viruses were the same; however, their proportions differed (Figure 3A). The common viruses that appeared > 0.1% in the viral community were 302 vOTUs, which accounted for 80.54% of the total relative abundances. The predominant viruses were *Acanthamoeba polyphaga moumouvirus*, *Orpheovirus IHUMI-LCC2*, *Megavirus chiliensis*, *A. polyphaga mimivirus*, and *Cafeteria roenbergensis virus BV-PW1*. These viruses belong to *Mimiviridae* and *Orpheoviridae*. (Figure 3B). These viruses typically infect eukaryotic plankton [30]. We investigated the hosts of viruses belonging to known infectious families but found no viruses known to infect bivalves. However, members of the *Iridoviridae* family are known to infect amoebas, and recent studies suggest that the genera *Ranavirus* and *Megalocytivirus* may be associated with infections in various marine species [41]. Notably, particles resembling an irido-like virus have been observed in oysters, specifically *Crassostrea angulate* [42]. These findings suggest that not only the *Herpesviridae* but also *Iridoviridae* and *Poxviridae* may have pathogenic potential in scallops. A hypothesis explaining their dominance in the MGs is that the primary food sources of scallops are plankton of various sizes, which, when concentrated through filter feeding, may promote viral proliferation in the scallop intestines due to eukaryotic plankton viral infections. Bivalve tissues comprise complex systems with distinct microbiome compartments, including viral, bacterial, and eukaryotic communities [43]. Viruses within bivalves form specific communities in the microbiome [30,40], with external environmental factors remarkably influencing viral composition differences [44]. The results suggest that the viral community originates from seawater and is transmitted to scallops, closely associated with bivalve host lineages. For example, the abundance of species belonging to *Phycodnaviridae*, which have a strong relationship with phytoplankton, observed in *C. farreri* suggests that they may have entered the intestine through filter feeding of the bivalve. Moreover, these predominant viruses may favour scallops as their preferred habitat. Filter-feeding bivalves serve as potential reservoirs for disseminating infectious marine viruses [30]. The viruses within bivalves are not random but inhabit specific populations. Imbalance in the host’s health and environmental conditions can lead to disease and death in the host [44,45].

**Figure 4:**
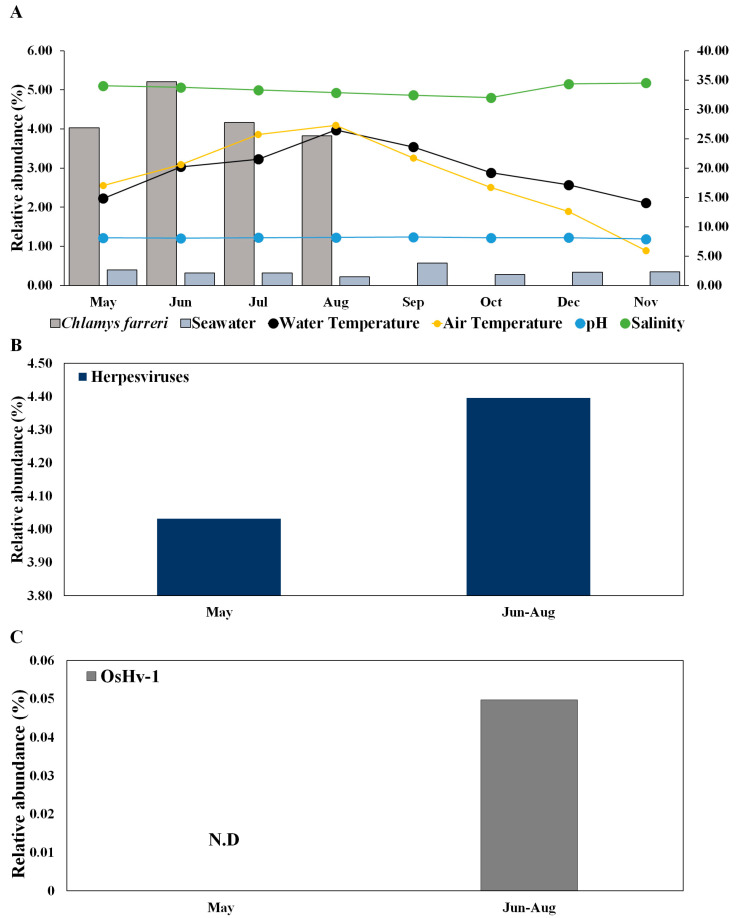
Change in the relative abundance of herpesviruses in *Chlamys farreri* and seawater along with environmental factors (**A**) and comparison between “May” group and “June to August” group of herpesviruses (**B**) and *Ostreid herpesvirus 1* (**C**) in Chlamys farreri. There was no significant difference between the two groups using the Wilcoxon test in (**B**,**C**).

Herpesviruses infect scallops and have high host specificity for these organisms [22,23]. In *C. farreri* and seawater, herpesviruses were detected at 36 and 17 vOTUs, respectively (Table S3). The mean relative abundances of *Herpesviridae* in *C. farreri* and seawater were 4.30% and 0.34%, respectively. The prevalence of *Herpesviridae* increased in the “June to August” group compared to in May (Figure 4A,B), suggesting that the infectivity and transmissibility of herpesviruses escalate with rising water temperatures [46]. While research on marine herpesviruses in bivalves is commonly conducted in the laboratory [47], field studies on viral immunology, specific primers, and bivalve microbiomes are scarce in marine ecological studies. Investigating the environmental interplay between scallops and seawater will yield crucial insights into infection transmission and potential infectious viruses. Furthermore, the emergence and activity of any virus can considerably affect disease progression in its host and reflect the growth and overall health status of the host [32].

Herpesviruses are particularly increased during summer mass mortality [18,27,48,49]. Herpesviruses were not detected in seawater, but these viruses detected in *C. farreri* exhibited high substrate specificity and were ubiquitously distributed throughout the study area. This study provides novel insights into the significant associations between *C. farreri* and seawater in terms of ecological emergence. OsHV-1 occurs primarily in response to elevations in temperature and light intensity caused by increased seawater temperature [19]. OsHV-1 is known to infect bivalves, which has negative implications for aquaculture [28,50,51]. In addition to OsHV-1, Abalone herpesvirus Victoria/AUS/2009 emerged. These species are known to infect bivalves such as abalone, and our results suggest that they emerged in May and may be potential infectious viruses [52]. Although the roles of scallops as reservoirs of infection for subsequent mortality outbreaks remain unclear, OsHV-1 was observed in *C. farreri* during the summer months (July), and all *C. farreri* died in September owing to the combined effects of high temperature and infection with OsHV-1 in this study (Figure 4C).

In conclusion, in this study, we suggest that *C. farreri* is a reservoir of high viral diversity, and its mass mortality is affected by the environment and viral infections in mass farming [53]. The detection of bacteriophages strongly suggests the presence of specific bacteria in the gut of scallops. Since bacteriophages have a close relationship with their bacterial hosts, this indicates that these bacteria may be actively involved in the gut microbiome of the scallops [54]. Furthermore, due to the filter feeding behaviour of scallops, various microorganisms and eukaryotic plankton (e.g., bacteria and algae) can accumulate from seawater, which may have significant impacts on the physiological or ecological status of the scallops [30]. Bacteriophages may also alter this gut ecosystem or interact with specific bacteria, potentially leading to pathological or health-related changes [55]. Furthermore, discovering various potential algae viruses highlights their possible role as infection hotspots. Therefore, studies on the structure and function of viral communities in bivalves will significantly enhance our understanding of their role in microbial community regulation, disease transmission, and the potential to protect and restore coastal ecosystems. This study preliminarily identified specific viral characteristics in *C. farreri* by comparing viral communities between scallops and seawater. However, further studies are planned to conduct an in-depth viral characterisation, including identifying specific viruses and their genetic makeup, exploring virus–host interactions such as disease mechanisms and potential control strategies, as well as investigating the ecological impact of viral infections in bivalves and their food sources.

## Data Availability

The raw sequencing data (Fastq files) are available on the Sequence Read Archive public database of NCBI under the project number PRJNA1149925 (in scallops) and PRJNA1131106 (in seawater).

## Supplementary Materials

The following supporting information can be downloaded at: https://www.mdpi.com/article/10.3390/pathogens13110935/s1, Table S1: Size meausrment of *Chlamys farreri* (Width, Height and Weight); Table S2: Summary of metavirome data (the total bases, reads, GC (%), Q20 (%) and Q30 (%), contigs); Table S3: Presented lists of herpesviruses and their relative abundance (%) found in *Chlamys farreri* and seawater, along with species identification information (Average Contigs and Average Similarity).
